# The translocator protein (TSPO) is prodromal to mitophagy loss in neurotoxicity

**DOI:** 10.1038/s41380-021-01050-z

**Published:** 2021-03-04

**Authors:** Michele Frison, Danilo Faccenda, Rosella Abeti, Manuel Rigon, Daniela Strobbe, Britannie S. England-Rendon, Diana Cash, Katy Barnes, Mona Sadeghian, Marija Sajic, Lisa A. Wells, Dong Xia, Paola Giunti, Kenneth Smith, Heather Mortiboys, Federico E. Turkheimer, Michelangelo Campanella

**Affiliations:** 1 Department of Comparative Biomedical Sciences, The Royal Veterinary College, University of London, Royal College Street, London, United Kingdom; 2 Ataxia Centre, Department of Clinical and Movement Neurosciences, UCL Queen Square Institute of Neurology, Queen Square London, United Kingdom; 3 Department of Biology, University of Rome TorVergata, Via della Ricerca Scientifica, Rome, Italy; 4 Department of Neuroimaging, Institute of Psychiatry, King’s College London, Camberwell, United Kingdom; 5 Sheffield Institute for Translational Neuroscience (SITraN), University of Sheffield, Sheffield, United Kingdom; 6 Department of Neuroinflammation, UCL Queen Square Institute of Neurology, London, United Kingdom; 7 Imanova Limited, Centre for Imaging Sciences, Imperial College London, Hammersmith Hospital, London, United Kingdom; 8 University College London Consortium for Mitochondrial Research, London, United Kingdom; 9 Present Address: MRC Mitochondrial Biology Unit, Cambridge Biomedical Campus, Cambridge, United Kingdom

**Keywords:** Biochemistry, Cell biology, Predictive markers

## Abstract

Dysfunctional mitochondria characterise Parkinson’s Disease (PD). Uncovering etiological molecules, which harm the homeostasis of mitochondria in response to pathological cues, is therefore pivotal to inform early diagnosis and therapy in the condition, especially in its idiopathic forms. This study proposes the 18 kDa Translocator Protein (TSPO) to be one of those. Both in vitro and in vivo data show that neurotoxins, which phenotypically mimic PD, increase TSPO to enhance cellular redox-stress, susceptibility to dopamine-induced cell death, and repression of ubiquitin-dependent mitophagy. TSPO amplifies the extracellular signal-regulated protein kinase 1 and 2 (ERK1/2) signalling, forming positive feedback, which represses the transcription factor EB (TFEB) and the controlled production of lysosomes. Finally, genetic variances in the transcriptome confirm that TSPO is required to alter the autophagy–lysosomal pathway during neurotoxicity.

## Introduction

Prevalence of psychiatric symptoms in primary mitochondrial diseases are common [1, 2] and debilitating [3]. Secondary mitochondrial diseases are equally affected, and Parkinsonism is a vastly acknowledged example [4–7]. Centred around a progressive age-related loss of dopaminergic circuits -responsible for bradykinesia and tremor-, Parkinsonism and PD display non-motor features ranging from depression, gambling addiction, and dementia. The symptomatology of PD varies substantially between patients, with mechanisms that remain ill-defined. One useful tool to insight those and stratify patients from a mitochondrial perspective is the Translocator Protein (TSPO) [8, 9]. This multidrug-binding protein, which is resident on the Outer Mitochondrial Membrane (OMM), has been studied for over two decades as a diagnostic marker of neurotrauma and target for anxiolytic therapy [10, 11]. Recently, evidence demonstrating the increased TSPO ligand binding in PD patients [12] made us query whether the protein had a causal role in the mitochondrial dysfunction underlying the condition. Clinical positron emission tomography (PET) imaging of patients with PD, dementia with Lewy bodies [13], and progressive supranuclear palsy [14] have revealed a complex but clinically valuable positive correlation between TSPO and the heterogeneous neurological damage [12]. Even though basally expressed at high levels only within steroid producing tissues [15, 16]_,_ TSPO is promptly inducible in cell lineages of the immune systems, including those residents in the CNS such as microglia [17–19]. In those, neurological stress induces a rapid and localised increase in TSPO [20–22]. However, the same occurs in neurons [18, 23–27], measurable via its targeting compounds [8, 19, 28, 29].

Previously we reported that TSPO prevents mitochondrial ubiquitination and impairs mitophagy [30], speculating that this anti-mitophagy function could be replicated in diseases. Even though PD is multifactorial, the accumulation of defective mitochondria is considered one of its hallmark [31, 32] driven by impairments in their autophagic degradation [7, 33, 34]. Studies of the hereditary forms of PD have indeed unveiled mutations in PTEN-induced kinase 1 (*PINK1*) [35] and the RBR E3 ubiquitin protein ligase Parkin (*PARK2*) genes [36], associated with a failure of mitochondrial autophagy as one of the causative mechanisms [37–39]. However, the PINK1-Parkin pathway of mitophagy requires potent toxins, like ETC uncouplers (e.g. FCCP), to be observed in vitro while disposable for mitophagy in vivo [40, 41]. In addition, recent studies have suggested physiological functions for the PINK1-Parkin pathway, which go beyond mitophagy, such as inflammation [42, 43] and regulation of mitochondrial protein import [44–46]. The selection and isolation of damaged mitochondria from the network require the essential ubiquitination of OMM proteins to consent their removal via the autophagosomal-lysosomal pathway [34, 38, 47, 48].

Based on this evidence, we set out to probe whether neurotoxin-induced mitochondrial damage is associated with the upregulation of TSPO and deregulation of mitophagy. We did so aware that: (i) TSPO expression is driven by activation of the extracellular signal-regulated protein kinases (ERKs) [49], and (ii) the mitogen-activated protein kinase (MAPK)/ERK signalling pathway is a relevant event during neurotoxicity and particularly PD [50]. The phosphorylated form (pERK1/2) is indeed found in Lewy Body aggregates and autophagosomes of idiopathic PD patients [51]. Parkinsonian neurotoxins [6-OHDA, rotenone, and N-methyl-4-phenylpyridinium (MPP+) particularly] induce ERK phosphorylation following an increase in Reactive Oxygen Species (ROS), which actively confer neurotoxicity by inducing cytotoxic autophagy [52] and impairing mitochondrial biogenesis [53]. Therefore, activated ERK may play a role in mediating mitochondrial dysfunction during PD-like neuronal injury [54] and TSPO high expression which is indeed driven by ERK [8].

Here, we demonstrate that TSPO is a factor in the mitochondrial aetiology of neurotoxicity by operating at multiple levels to impair the autophagosome-lysosomal pathway triggered by PD toxins. Therefore, TSPO emerges as a pro-pathological conduit for mitochondria, which results in several distinctive signatures of the transcriptome.

## Materials and methods

### Animal experiments

6-OHDA lesioned rat brain sections for immunohistological analysis were prepared as previously described [55]. Briefly, the rats (male, Sprague Dawley, 250 ± 25 g, Harlan, UK) were group-housed at standard conditions, and all experiments were conducted under the Home Office Animals (Scientific procedures) Act, UK, 1986 and were approved by the King’s College London ethical review committee (project licence P023CC39A, D Cash). The lesioning with 6-OHDA was achieved by stereotaxically administering 12 µg of 6-OHDA into the medial forebrain bundle. The success of lesioning was verified by apomorphine-induced rotations (>100 in 60 min), and the animals sacrificed 3 weeks after 6-OHDA by transcardiac perfusion and fixation with heparinised saline, followed by 4% buffered paraformaldehyde. PET imaging was carried out on a separate set of animals in a separate facility. Male Sprague-Dawley rats (Charles River, UK) weighing ca. 250 g, were housed in groups of three per cage under a controlled temperature (21 + 1 °C), relative humidity (60%) and a 12 h light/dark cycle, with access to food and water ad libitum.

### Surgery and imaging

Under recovery anaesthesia (isoflurane), rats were injected with 6‐OHDA. Twelve µg 6‐OHDA free base in 4 µl 0.1% ascorbic acid in 0.9% saline (*n* = 4) or vehicle (control *n* = 2) was injected stereotactically [56] into the left medial forebrain bundle (mfb) (−2.2 mm A/P, +1.5 mm L/M coordinates from bregma; −7.9 mm D/V from dura), as previously described by the authors [57]. Nine days post-lesion rats received an i.v. bolus of [^11^C]PBR28. Subsequently, two of the four lesioned rats underwent a PET:CT scan. [^11^C]PBR28 was prepared by ^11^C-methylation of the corresponding *O-desmethyl* precursor using [^11^C]methyl iodide. The specific activity was 289 GB q/umol. For imaging studies, the femoral vein and artery of SD rats were cannulated under terminal isoflurane anaesthesia prior to [^11^C]PBR28 administration. A dynamic PET:CT scan (INVEON DPET/MM PET:CT, 60 min) was carried out and reconstructed with 2D FBP (CT attenuated with scatter correction). Blood samples were collected to obtain a metabolite-corrected input function. Regions of interest (ROI) were drawn on the lesion site and an equivalent contralateral area. Time activity curves (TACs) were derived, and the volume of distribution (V_T_) was estimated. After 60 min, all rats were exsanguinated; blood and plasma were collected, and brain regions were removed and washed in chilled saline, and the associated radioactivity was determined in all samples by a gamma counter. For [^3^H]PBR28 radiography, striatal and substantia nigral sections (4.52–4.80 mm from Bregma) were cut onto poly-l-lysine coated slides and incubated with 0.5 nM [3H]PBR28 for 60 min. To determine non-specific binding, slides were incubated with 0.5 nM [3H]PBR28 with an excess of 10 µM PK11195 for 60 min. Slides were washed and allowed to dry in a cool stream of air. Slides were covered by a scintillation sheet and counted for 24 h per slide. Quantification of the autoradiography images was carried out using ImageJ, by comparing the values extracted from each ipsilateral hemisphere to respective contralateral Control. Each image was separately assessed using the Max Entropy algorithm in the area of pixels above threshold.

### Cell culture and transfection

SH-SY5Y cells were obtained from ATCC and were maintained in Dulbecco’s Modified Eagle’s medium (DMEM) (Thermo Fisher, 11995073) supplemented with 10% Foetal bovine serum (FBS) (Thermo Fisher, 10082-147) and 1% penicillin/streptomycin (P/S) (Thermo Fisher, 15140122). To induce differentiation, a serial treatment combination of reduced medium (DMEM + 0.5 % FBS and 1 % P/S) for 10 days, with the first 3 days in 15 μM retinoic acid (RA; Sigma, R2625) and the successive 7 days of 0.5 ng/mL BDNF (Sigma, B3795). Transfection was carried out using a standard Calcium (Ca^2+^) phosphate method, as described previously [58]. In experiments containing +TSPO and −TSPO conditions together, the RNAi samples (nsc and −TSPO) were normalised to the mean of the mock transfected samples. Treatments were initiated 48 h post-transfection. Plasmid transfection was used to induce expression of exogenous proteins, over-expression of selected proteins or downregulation of TSPO via RNA interference (SI02777061, Qiagen; sense: CAUCUUCUUUGGUGCCCGATT; antisense: UCGGGCACCAAAGAAGAUGGG). Plasmid list: scrambled RNA (scrRNA) (1027281, Qiagen), non-silencing control (nsc); mTSPO - pCR™ 2.1-TOPO® TA vector with gene D21027, Parkin-YFP (Addgene, 23955), LC3-GFP (Addgene, 210730), ubiquitin-GFP (Addgene, 11928), CFP (Addgene, 1179), GFP (Addgene, 1150), mtGFP – generous gift from Prof Rizzuto, mtRFP (Evrogen, FP147), dsRed (Addgene, 11151). Stable TSPO knockdown was achieved using the pGIPZ shRNA vector: clone ID V3LHS_331646, target sequence: 5′-TGAGTGTGGTCGTGAAGGC-3, purchased from Open Biosystems (Huntsville, AL, USA). Non-silencing shRNA vector sequences (mature antisense): V3LHS_331648: 5′-ACGCAGTAGTTGAGTGTGG-3′ V3LHS_331650: 5′-TCTGCAGGCCGGCGTACCA-3′. After transfection, cells were maintained for 2 weeks in media supplemented with 3 μg/ml puromycin (SERVA Electrophoresis GmbH) to select transfected (GFP-positive) cells. TSPO KO SH-SY5Y cells were generated by transient transfection with GeneArt™ CRISPR Nuclease Vector with OFP Reporter Kit (Thermo Fisher, A21174), using a gRNA (5′-GGGCACGCTCTACTCAGCCA-3′) targeting exon 2. Fibroblasts were obtained from Coriell Cell Repository (coriell.org) via MTA with HM; patient samples and Control samples were age-and sex-matched (details given in Table 1). Fibroblasts were cultured and maintained in EMEM (Gibco) as previously described [59]. Fibroblasts were passage matched for experiments and used between passages 10 and 15.

**Table 1:** The table details the information on the participant samples used for this study.

Code	Status	Mutation	Age at biopsy (yrs)	Sex
GM02189	Control		63	M
GM029510	Control		55	F
GM09400	Control		61	F
GM07924	Control		52	M
ND31618	Parkin mutant	ARG42PRO	63	F
ND30171	Parkin mutant	ARG42PRO/EX3DEL	54	M
ND40078	Parkin mutant	ARG275TRP/ARG275GLN	51	F
ND37732	Parkin mutant	EX3 40 BP DEL/EX4DEL	63	F

### Immunocytochemistry

Cells were initially plated on 22 mm glass coverslips at a 30% confluency and collected 48–72 h after transfection. At treatment completion, cells were washed once with 0.01 M Phosphate Buffered Saline (PBS 1X) and fixed with 4% paraformaldehyde (PFA) with a 10 min incubation. Cells were then washed three times for 5 min each with PBS 1X and permeabilised with a 0.5% Triton-X100/PBS 1X solution for 10–20 min. After another round of washes, cells were blocked for 1 h in a solution of PBS 1X containing 10% v/v goat serum (Thermo Fisher, 16210064) and 3% w/v BSA. Primary antibody was incubated overnight at 4 °C in blocking solution at the following concentrations: 1:250 ATP5B (Abcam, ab14730), 1:250, TFEB (Cambridge Bioscience, A303-673A) or 1:100 Tau (Abcam, ab32057). After three washes, the secondary antibody was incubated for 1 h at a 1:500 dilution of the following secondary antibodies in blocking solution: anti-mouse goat IgG Alexa 488 (Abcam, A11001), anti-rabbit donkey IgG Alexa 568 (Abcam, A10042), anti-mouse goat IgG Alexa 633 (Abcam, A21050). After 1 h at room temperature away from light, the coverslips were washed and mounted onto glass slides using a 4′,6′-diamidino‐2-phenylindole (DAPI)-containing mounting medium (ab104139). The coverslips were then imaged on a Leica SP5 confocal microscope.

### Western blotting

Lysates were either made from mitochondrial fractions and whole cells. Fractions were collected in sucrose buffer (50 mM sucrose, 10 mMKCl, 20 mM HEPES, 1.5 mM MgCl2, 1 mM EDTA, 1 mM EGTA [Sigma, E8145], protease inhibitor [Roche, 4693132001], pH 7.4) on ice. To break the plasma membrane, the cell suspension was passed through a 26-gauge needle (Sigma, Z192392) ~30 times. Separation of the mitochondrial and cytoplasmic fractions was performed using differential centrifugation, as shown previously [60]. Primary antibodies: ACTB (Abcam, ab8826) 1:5000, ATP5B (Abcam, ab14730) 1:5000, ERK1/2 (Cell signalling, 9102S) 1:1000, pERK1/2 (Y202/Y204) (Cell signalling, 9101) 1:500, SDHA (Abcam, ab109865), TSPO (Abcam, ab109497) 1:4000, USP30 (Enzo, MBL-PW0975) 1:1000, VDAC1 (Abcam, ab14734) 1:2000, 1:10000 Vinculin (Abcam, ab129002) and 1:1000 TFEB (Cambridge Bioscience, A303-673A). Imaging was carried using an ECL based method (GE, RPN2133) on a a Bio-Rad ChemiDoc MP Imaging System, following incubation for 1 h with either anti-rabbit conjugated HRP 1:4000 (Dako, P0447), or rabbit anti-mouse conjugated HRP (Dako, P0448), or goat anti-rat IgG conjugated HRP 1:4000 (Abcam, ab97057). Densitometric analysis was performed using the ImageJ software. Values were normalised to ACTB loading control for whole-cell lysates and ATP5B for mitochondrial fractions.

### Immunohistochemistry

Following PBS washing, slices were incubated at 80 °C for 30 min in citrate buffer: 10 mM sodium citrate dehydrate (Sigma, W30200), 0.05% Tween‐20 (Sigma, P7949), pH 6.0. Washes were followingly carried out in PBS-T (0.05% Tween-20 in PBS). Permeabilization was carried out for 15 min at 4 °C in 20 mM HEPES, 300 mM sucrose (Sigma, 59378), 50 mM NaCl, 0.05 % Triton‐X100 (Sigma, T8787), 3 mM MgCl_2_, 0.05% sodium azide (Sigma, 71290). Blocking – 1 h at room temperature in 10% horse serum (Thermo Fisher, 16210064), 1% w/v bovine serum albumin (BSA) (Sigma, A2153) in PBS 1X. Primary antibody incubation was carried out overnight at 4 °C first using a 1:150 dilution of rabbit TSPO antibody (biorbyt, orb5845) and the next day, after washing, using rat DAT antibody (Millipore, MAB369), 1:150 in blocking solution. Secondary antibodies: anti-rat goat IgG Alexa 555-conjugated (abcam, ab105158), anti-rabbit goat IgG Alexa 488-conjugated (Invitrogen, A11008) at 1:200 in blocking solution for an hour at RT, while shaking. Slices were washed 4–5 times in PBS-T 1X and three times in PBS 1X, before a 5 min incubation with 600 nM DAPI solution (D1306, Invitrogen). Excess DAPI was cleared with three 5 min washes in PBS 1X and slices were then mounted on glass slides (VWR, 631-0908) using a polyvinyl alcohol mounting medium containing 1,4-diazabicyclo[2.2.2]octane (DABCO®) (Sigma, 10981), covered with 22 mm glass coverslips. The slices were then imaged on a confocal Leica SP5 with a 63x lens. TSPO intensity was gathered from DAT stained projections within the globus pallidus and entopeduncular nucleus of multiple stained slices (*n* = 3). Equal numbers of neurons from each slice and each area were compared.

### Quantitative PCR

Quantitative reverse-transcription PCR (qRT-PCR) was carried out with a standard calibration curve containing serial dilutions of each cDNA transcript for each separate gene. The mRNA isolation step was carried out using the RNeasy Plus Mini kit (Qiagen, 74134), as per manufacturer’s instructions. cDNA synthesis was carried out using the QuantiNova™ Reverse Transcription Kit (Qiagen, 205411), in an RNase-free environment. Only RNA samples with 260/280 nm of 2.0 ± 0.2 were used. Real-time PCR, executed with the QuantiNova SYBR Green PCR kit (Qiagen, 208052), was used to amplify the standards and then quantify the sample’s mRNA expression. Using 96-well PCR plates (Thermo Fisher, AB0800W), covered by adhesive seals (4titutde, 4ti-0565), triplicates of each sample were mixed with SYBR green dye and appropriate primers. Details: MAOB (proprietary, qHsaCID0017743), TSPO (proprietary, qHsaCED0037846), USP30 (S: GGGATTATAGACCGGACACTA, AS: CACAAGCCCTTTTCTACGCT); 18S (S: CGCGGTTCTATTTTGTTGGT, AS: AGTCGGCATCGTTATGGTC). The 96-well plate was placed into a CFX Connect™ Real-Time PCR Detection System, and 40 cycles of denaturation (5 s, 95 °C) and extension (10 s, 60 °C) were executed.

### Redox analysis

Dynamic analysis of cellular redox was carried out using four different live cell imaging dyes, that detect specific molecular species: cytoplasmic superoxide is analysed using DHE (Life Technologies, D11347); mitochondrial superoxide is detected with mitoSOX™ Red (Life Technologies, M36008); global cellular hydrogen peroxide (H_2_O_2_) and peroxyl radicals (HO_2_) are measured using DCFDA (Sigma, D6883) while unbound reduced glutathione (GSH) is assayed with MCB (Life Technologies, M-1381MP). Cells were cells co-transfected with an appropriate fluorophore that would not interfere with the fluorescence of the dye. All dyes were administered to the recording medium (RM) onto cells plated on coverslips, within Attafluor® metal cell chambers (Molecular Probes™, Thermo fisher, A-7816). RM composition is: 5.6 mMKCl (Sigma, P9333), 10 mM D-(+)-Glucose (Sigma, G7528), 10 mM HEPES (Sigma, H4304), 4.2 mM NaHCO_3_ (Sigma, 56297), 138 mM NaCl (Sigma, S5886), 2.6 mM CaCl_2_ (Sigma, C7902), 1.2 mM NaH_2_PO_4_ (BDH,1024940), 1.2 mM MgCl_2_ (BDH, 10149), pH 7.4.

DHE (10 μM), mitoSOX (10 μM), and DCFDA (15 μM) were diluted in RM and imaged onto a Zeiss LSM510 confocal microscope. MCB was used at final concentration of 2.5 μM in RM. Brightness controlling settings were maintained consistently within the experiment for all techniques.

### mtKeima measurement

To analyse the delivery of mitochondrial content to the lysosome, we used a plasmid vector expressing mt-Keima ([Amalgaam, AM-V0251]). Cells were transfected with mtKeima, treated, and successively imaged on a Leica SP5 microscope while in RM buffer at 37 °C. Using ImageJ, ratio pictures of 586/440 nm excitation were created, and the number of pixels emitting only red signal over the total number of pixels per cell was used as mitophagy index.

### Imaging of mitochondria

To image the lysosomal population, cells were transfected with DsRed and TSPO siRNA/nsc, treated in culture media, stained with 100 nM LysoTracker™ DND-22 (Thermo Fisher, L7525) for 30 min and then imaged on a Leica SP5, in RM. Their number or volume was quantified on ImageJ, using the “3D object counter” plugin. Mitochondrial morphology was assayed using cells transfected with a mitochondria-targeted GFP (mtGFP), utilising only cells with neurites. Aspect Ratio (AR), a measure of elongation, and Form Factor (FF), the inverse of circularity and a measure of branching, were calculated on the whole mitochondrial network, with ImageJ software. ΔΨ_m_ was measured using tetramethylrhodamine methyl ester (TMRM). Fluorescence intensity was directly measured after incubation of 50 nM TMRM in RM for 30 min on a Zeiss LSM510 confocal microscope. To measure basal membrane potential, only mitochondrial ROIs were used, while the rate of MPP(^+^)-induced depolarisation was measured using live recordings of whole-cell ROIs.

### Transcriptome data analysis

Using the CRISPR/Cas9 system, we generated a line of SHY-SY5 ablated for TSPO gene (SHY-TSPO KO). This and the control line were exposed to MPP + before extracting the total RNA. Total RNA samples from TSPO WT and KO cells were extracted using TRIzol reagent (Invitrogen; 374 Thermo Fisher Scientific, Waltham, MA, USA) and then quantified with an Agilent 2100 bioanalyzer. The quantified RNA samples were used for NEBNext® rRNA-depleted (Human/Mouse/Rat) stranded library preparation. Library preparation and RNA sequencing was conducted at the UCL Genomics. Libraries were prepared using non-strand specific Illumina TruSeq Sample Preparation Kits followed by Illumina sequencing. FASTQ files were aligned using TopHat and Cufflinks. Normalisation and differential analyses were carried out using R software Bioconductor [61] package DESeq2 [62], and gene set enrichment analysis (GSEA) was carried out using EGSEA [63].

### Assessment of cell viability

Cell viability was measured using propidium iodide (PI, Sigma, P2667-2OTST). Cells were co-transfected with GFP in six well plates. After treatment, wells were washed with PBS once and successively incubated for 10 min with 3 µg/ml of PI in RM. Three washes with PBS were used to remove excess stain. The cells were then maintained in RM and imaged immediately using the Leica DMIRB inverted fluorescence microscope. The proportion of PI-positive cells within the transfected population was used as a measure of viability. The trypan blue exclusion method (100, 300 µM, Sigma) was also enrolled, and cells counted under the KOVA® Glasstic Slide with Counting Grids.

### Data analysis

Data are presented as mean ± standard error of the mean. Statistical analysis was performed using R software (version 3.0.2). All analyses were carried out in a standardised manner. In summary, to determine whether the sample distributions were normal, a Shapiro–Wilk test was performed for sample groups with *N* < 50; for sample groups with *N* ≥ 50, a histogram and a quantile-quantile were employed. For experiments with two normally distributed conditions, a Welch’s *t*-test was performed. If the experiment included multiple conditions, a one-way analysis of variance (ANOVA) tests was performed to address if the variance between samples was smaller than between conditions, (i.e. vehicle-treated ± TSPO cells to MPP+ treated). Comparisons between conditions were carried out using a pairwise *t*-test, utilising Holm’s correction. If any sample failed the normality distribution tests, a Wilcox test was used to compare two conditions, a Kruskal–Wallis test was used to compare groups of conditions and a Wilcoxon rank sum tests with Holm’s multiple correction for pairwise comparisons. The N numbers utilised indicate the number of biological repeats. In assays that compare bulk groups of cells, i.e. WB or cell viability, 3 or 4 separate sets of experiments (and treatments) were compared, with technical repeats (multiple measurements of the same biological sample) averaged out. In imaging experiments, two separate treatments and transfections were carried out, and the number of cells analysed per condition is reported as N. In the PET and autoradiography experiments, the N number indicates the total number of animals used per condition. Statistical significance is indicated as follows: ‘*’ – *p* < 0.05; ‘**’ – *p* < 0.01; ‘***’ – *p* < 0.001.

## Results

### TSPO expression increases in Parkinson’s disease

**Fig. 1:**
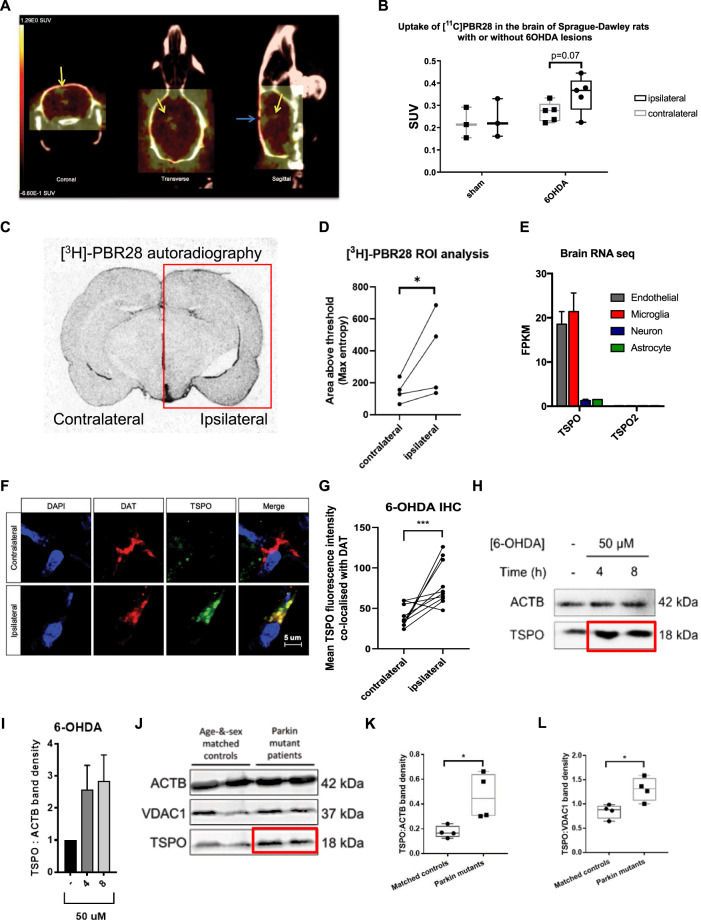
In vivo and in vitro upregulation of TSPO by Parkinsonian toxins. **A** A representative PET image of [^11^C]PBR28 uptake in the brain of a Sprague-Dawley rat subjected to a unilateral injection of 6-OHDA for 9 days. Yellow arrows indicate representative areas of high uptake; the stereotaxic drill hole is indicated in the sagittal view by a blue arrow. **B** Bar charts displaying the average [^11^C]PBR28 uptake in the whole hemisphere of 6-OHDA lesioned rats (*n* = 5), with contralateral control hemisphere, and of sham samples (*n* = 3), subjected to an injection of saline. **C** Representative autoradiography section of unilateral 6-OHDA lesioned rats displaying [^3^H]-PBR28 uptake, with a (**D**) threshold-based image quantification of [^3^H]-PBR28 signal comparing whole ipsilateral to contralateral hemispheres. **E** Comparison of TSPO expression in endothelial, microglia, neuron, and astrocyte tissues using a public mRNA database [64]. **F** Representative immunohistological images of coronal sections (contralateral and ipsilateral origin) of the 6-OHDA-treated rats stained for DAPI, TSPO, and DAT. **G** Image-based quantification of co-localised TSPO signal intensity in DAT-stained neurons from different midbrain sections (*n* = 10 cells per condition). **H** Representative bands of WB of TSPO and loading control ACTB in whole SH-SY5Y cell lysates, under the administration of 50 µM 6-OHDA for 4 or 8 h, with, in (**I**), its ACTB-normalised band density (*n* = 4). **J** Representative image from a SDS-PAGE analysis of TSPO expression, controlled against ACTB and the mitochondrial VDAC1, in fibroblasts of Parkin mutant PD patients, with respective age-&-sex-matched controls. Band density quantification of TSPO expression normalised (**K**) the loading control ACTB and (**L**) to the mitochondrial control VDAC1 (*n* = 4).

Our analysis began by assaying the accumulation of TSPO in vivo using second-generation ligands for the protein in a PD rat model. Animal brains were scanned via PET 9 days after unilateral injection of 6-OHDA. Based on the data collected, a heat map representing the binding of the radiolabelled, high-affinity [16, 29] TSPO ligand [^11^C]PBR28 was generated (Fig. 1A). This effect was quantified via biodistribution analysis (Fig. 1B), indicating a marked upregulation of [^11^C]PBR28 uptake in the brains of 6-OHDA-injected animals when compared to the sham-injected group. Moreover, the extent of [^11^C]PBR28 uptake after 6-OHDA injection was significantly higher in the ipsilateral brain hemisphere of treated animals than in the contralateral one. Further analysis of coronal sections of the central nervous system (CNS) of rats subjected to the same treatment was run via autoradiography, using radiolabelled PBR28 (Fig. 1C). The quantification in 1D showed a generalised increase in radioactive signal in the ipsilateral hemisphere of 6-OHDA-injected animals, confirming the PET’s trend observed previously. Even though the accumulation of TSPO is commonly acknowledged to be occurring in glial and microglial cells, neurons can express the protein too (Fig. 1E) [64]. In order to test if dopaminergic neurons can upregulate TSPO following treatment with neurotoxins, we carried out an immunohistological analysis of coronal brain sections of 6-OHDA-treated rats (*n* = 3). Different midbrain sections were selected, labelled with the nuclear stain 4′,6-diamidino-2-phenylindole, dihydrochloride (DAPI), and an immunoassay was run for TSPO and dopamine transporter (DAT) [65–67] (Fig. 1F, G). By looking at the globus pallidus and the entopeduncular nucleus, we found that dopaminergic neurons revealed a predominant co-localisation between TSPO and DAT-positive, and following treatments with the neurotoxins, TSPO was markedly overexpressed (Fig. 1F, G). Validation of TSPO upregulation in PD rat models and its neurotoxin-induced accumulation in dopaminergic neurons represented the basis for the next in vitro set of analyses, which began by exposing SH-SY5Y cells to 6-OHDA (50 μM, 4–8 h) and measuring TSPO levels via Western blotting (WB) (Fig. 1H, I). The analysis corroborated the results obtained both in vivo (Fig. 1A, B) and ex vivo (Fig. 1F, G). To further the relationship between PD-related mutations and increased TSPO expression, fibroblasts from PD patients (*n* = 4) bearing point mutations and/or exon deletions on the *PARK2* gene (Table 1) were characterised by significant TSPO over-expression when compared to age-and-sex-matched controls. (Fig. 1J–L).

### TSPO upregulation is the result of an ERK1/2-dependent transcriptional response

**Fig. 2:**
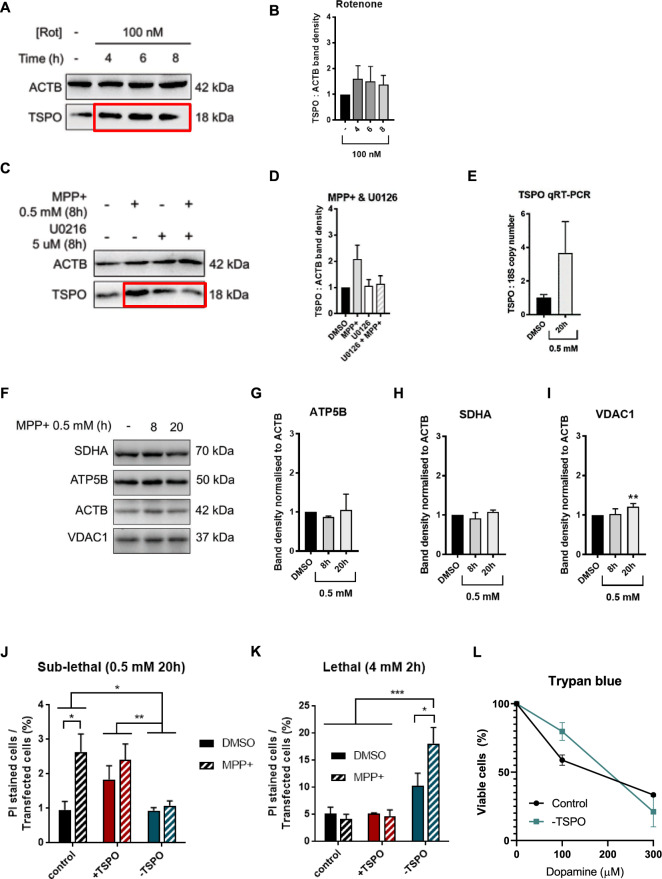
MPP+ induces an ERK-dependent transcriptional upregulation of TSPO. **A** Representative WB bands of SH-SY5Y cells treated with 100 nM Rot for 4, 6, and 8 h. Whole-cell protein lysates were probed for TSPO and β- actin (ACTB). **B** Average band densities were normalised to ACTB, (*n* = 3). **C** Representative WB bands of TSPO and ACTB in SH-SY5Y whole-cell lysates, following administration of 0.5 mM MPP+ for 8 h, in absence or presence of the MEK inhibitor U0126. TSPO band densities are given in (**D**) (*n* ≥ 6). **E** The mRNA levels of both *TSPO* and *18S* ribosomal RNA were measured by reverse transcription quantitative real-time PCR. TSPO absolute copy number was normalised over 18S (*n* = 3). **F** Representative SDS-PAGE of wild type SH-SY5Y cells treated (Control) for 8 and 20 h with 0.5 mM MPP+, immunoprobed for the IMM proteins ATP5B and SDHA, the OMM protein VDAC1 and the loading control ACTB. Band density quantification of the expression of (**G**) ATP5B, (**H**) SDHA, (**I**) VDAC1 normalised to ACTB (*n* ≥ 3). **J, K** PI stained-to-transfected cell ratio of cells treated with MPP+ dosages (0.5 mM for 20 h, 4 mM for 2 h) and expressing different TSPO levels (*n* = 3). **L** Trypan blue assay of Control and −TSPO cells following challenge with various dopamine concentrations (100, 200, and 300 µM). Results are presented as the ratio of living cells compared to non-treated.

Having observed that SH-SY5Y cells recapitulate the upregulation of TSPO observed in vivo, we administered two other PD-linked neurotoxins, chosen for their ability to induce ERK-dependent autophagy and Parkinsonism in vivo [68–71] (Table 2). By administering rotenone (Rot) (100 nM), we monitored the level of TSPO over time (4, 6, and 8 h) (Fig. 2A, B). The results showed that the increase in the expression of TSPO was achieved already at 4 h of treatment, and there were no significant differences between the time points tested (Fig. 2A, B). The second neurotoxin used, MPP+, had shown a more pronounced increase, compared to Rot, in TSPO previously [72–75], at a concentration of 0,5 mM in 8 h; therefore, we focused our subsequent analyses on this neurotoxin. The activation of ERK1/2 induces *TSPO* transcription via activator protein 1 (AP1) and signal transducer and activator of transcription 3 (STAT3).

**Table 2:** The table shows the three Parkinsonian neurotoxins used in the study, their chemical structures and their neurotoxic properties.

Acronym	Name	Chemical structure	Neurotoxicity
6-OHDA	6-hydroxydopamine		It is an hydroxylated analogue of dopamine
rotenone	rotenone		Inhibits the transfer of electrons from iron-sulfur centers in complex I to ubiquinone
MPP+	1-methyl-4-phenylpyridinium		Interferes with the oxidative phosphorylation in mitochondria by inhibiting complex I

To assess whether MPP+induced upregulation of TSPO expression was dependent on ERK1/2, we treated the cells with U0126 (5 μM, 8 h), a pharmacological inhibitor of mitogen-activated protein kinase (MEK)-induced ERK1/2 phosphorylation. As shown in Fig. 2C, D_,_ the co-administration of U0126 prevented the upregulation of TSPO in response to MPP+. At confirmation of the MPP+dependent increase of *TSPO* transcription, mRNA levels were assessed via qRT-PCR (Fig. 2E). SH-SY5Y cells were treated for a longer time (20 h), and the results showed a significantly higher expression of *TSPO* mRNA levels than untreated cells (Fig. 2E). Then we tested the effect of MPP+ (0.5 mM) at 8 and 20 h, looking at different mitochondrial proteins (Fig. 2F–H). Remarkably, the inner mitochondrial membrane proteins ATP synthase (ATP5B) and succinate dehydrogenase complex (SDHA) remained substantially unchanged (Fig. 2F–H). Perhaps unsurprisingly, the OMM protein voltage-dependent anion channel (VDAC1), to which TSPO is molecularly and functionally linked [60, 76, 77], increases its expression after 20 h of treatment by an average of 20% (Supplementary Fig. 1A–D). The hypothesis whereby the accumulation of TSPO represents a pro-pathological mechanism was corroborated further by the degree of cell death in SH-SY5Y in response to MPP+ using dosages (Fig. 2J, K) equally able to collapse the mitochondrial membrane potential (ΔΨ_m_) (Supplementary Fig. 2A–F). A low but sustained dose of the MPP+ damaged cells expressing TSPO (Control and +TSPO), leaving unharmed those in which the protein was down-regulated (-TSPO) (Fig. 2J). An opposite outcome was measured with the administration of MPP+ at a higher concentration, which was detrimental for neurons devoid of TSPO (Fig. 2K). Such an observation on cell death susceptibility was expanded further by using dopamine in toxic concentrations (100, 200, and 300 µM) and confirmed in differentiated SH-SY5Y cells (Fig. 2L).

### TSPO shifts the cellular redox balance of neurons

**Fig. 3:**
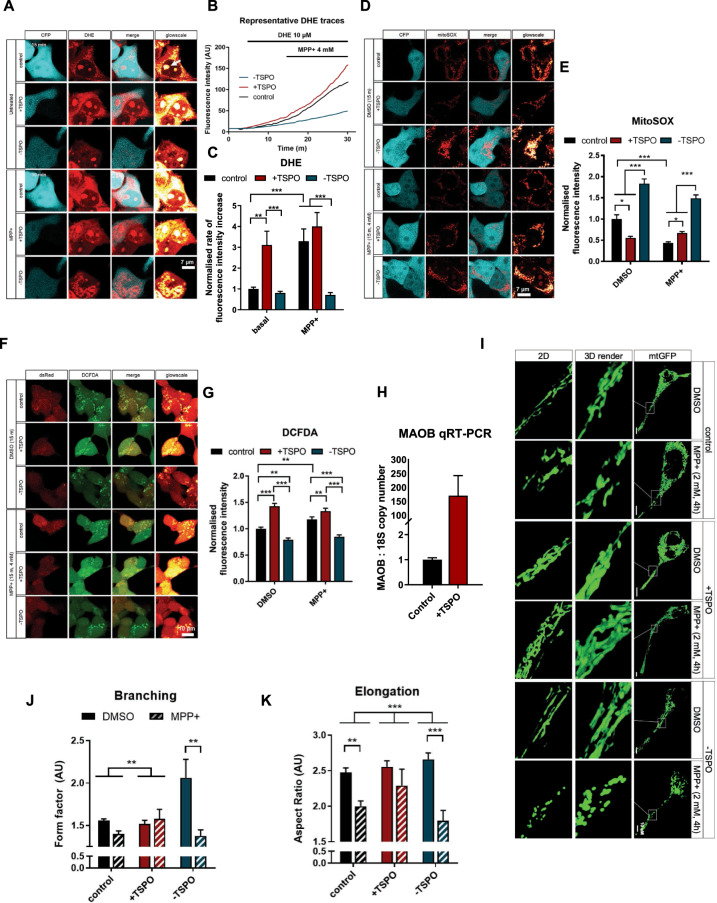
TSPO drives cellular redox-stress response triggered by the neurotoxin MPP+. **A** Representative DHE analysis images in ±TSPO cells transfected with CFP and treated with 4 mM MPP+ in acute (15 min). **B** Representative fluorescent traces and (**C**) linear DHE increment quantification for cells transfected with TSPO and treated with MPP+ (*n* ≥ 15 cells). **D** Representative images of mitoSOX^™^ staining of ±TSPO cells co-transfected with CFP and treated with 4 mM MPP+ shortly (15 min) before imaging. **E** Quantification of mitochondrial mitoSOX intensity from (**D**) (*n* ≥ 20 cells). **F** DCFDA analysis of cellular peroxyl radicals in cells transfected for TSPO and with the DsRed. 4 mM MPP+ treatment carried out for 15 min and (**G**) average DCFDA fluorescence count normalised to control (*n* ≥ 20 cells). **H** qRT-PCR of Control and +TSPO cells with primers for human MAOB. Absolute copy numbers were quantified, normalised to the loading control, the 18S gene, and normalised to transfection control. (*n* = 3). **I** Mitochondrial morphology analysis, using mtGFP, in ±TSPO cells treated with MPP+ (2 mM) for 4 h. Two mathematical image-based analyses were made: (**J**) form factor, a measure of branching, and (**K**) aspect ratio, a measure of elongation (*n* = 10 cells).

Subsequently, we adopted live-cell imaging tools to monitor different subcellular pools of ROS in SH-SY5Y cells mock-transfected (Control), transiently over-expressing TSPO (+TSPO) or down-regulated for the protein (-TSPO). We initially administered MPP+ in acute exposure (4 mM, 15 min) to cells loaded with dihydroethidium (DHE), which allows measuring the cytoplasmic pools of superoxide. Figure 3A–C showed that, at steady state, cytoplasmic O_2_^●-^ is heightened solely in +TSPO cells, with levels that are comparable to control cells after MPP+ treatment. While control cells underwent an increase in signal following MPP+ administration, −TSPO cells did not show any relevant change. By using the mitochondrially targeted, superoxide indicator MitoSOX™ (mitoSOX) as a read-out of mitochondrial ROS (mROS) (Fig. 3D, E), we instead observed that basal mitoSOX fluorescence emission was almost doubled in -TSPO cells when compared to control conditions, and that acute treatment with MPP+ (4 mM, 15 min) lowered its intensity. Oppositely, the over-expression of TSPO (+TSPO) counteracted the MPP+ increase in mitoSOX fluorescence (Fig. 3D, E). Analysis of global cellular hydroxyl radicals using 2ʹ,7ʹ-Dichlorofluorescin Diacetate (DCFDA) fluorescence showed the same trend observed with DHE (Fig. 3F, G). And thus, -TSPO cells display significantly lower accumulation of DCFDA fluorescence than Control or +TSPO cells and are protected from MPP+induced increase in ROS levels.

Control cells showed instead a statistically significant increase in global cellular hydroxyl radicals after acute treatment with MPP+ which is ineffective on +TSPO cells which feature a more oxidised basal redox balance at resting conditions. To monitor the effect of TSPO modulation on the antioxidant capacity of SH-SY5Y cells, we then analysed reduced GSH levels via monochlorobimane (MCB). The results showed that +TSPO cells are highly sensitive to the MPP+-induced generation of ROS, which causes a significant drop in the pool of reduced GSH that was not observed in either Control or -TSPO cells (Supplementary Fig. 1E–G). To further address the opposite relationship between cytoplasmic and mROS pools, we used superoxide scavengers to remove, selectively, ROS from each intracellular compartment. By employing the artificial antioxidant TEMPO or its mitochondrially targeted version: mitoTEMPO, we measured the compartment-specific pools of oxidised GSH, interpolated from the increase in MCB signal following scavenger treatment (Supplementary Fig. 1H). This revealed that -TSPO cells had oxidised GSH in both compartments, while +TSPO cells only in the mitochondria. In addition, mitoTEMPO does not shield +TSPO cells, like TEMPO, we infer that these cells are very susceptible to MPP+induced ROS in the cytoplasm.

Knowing that TSPO engages the Ca^2+^-dependent NADPH oxidases (NOX) [60, 78] to generate ROS, we tested whether monoamine oxidases (MAOs) [79–81] were involved in this redox rewiring. A qRT-PCR analysis revealed that +TSPO cells present vastly increased levels of MAOB (Fig. 3H). Finally, the pro-oxidant phenotype induced by TSPO over-expression is paired with a disorganised and dense mitochondrial network, which is more elongated and branched in -TSPO cells. However, TSPO depletion predisposes mitochondria to undergo a drastically more severe fragmentation after exposure to MPP+ (Fig. 3J, K). Therefore, TSPO over-expression induces an imbalance in redox production, which is likely to promote toxicity by neurotoxins leading to the assessment of mitophagy response in these conditions.

### The upregulation of TSPO de-ubiquitylates mitochondria

**Fig. 4:**
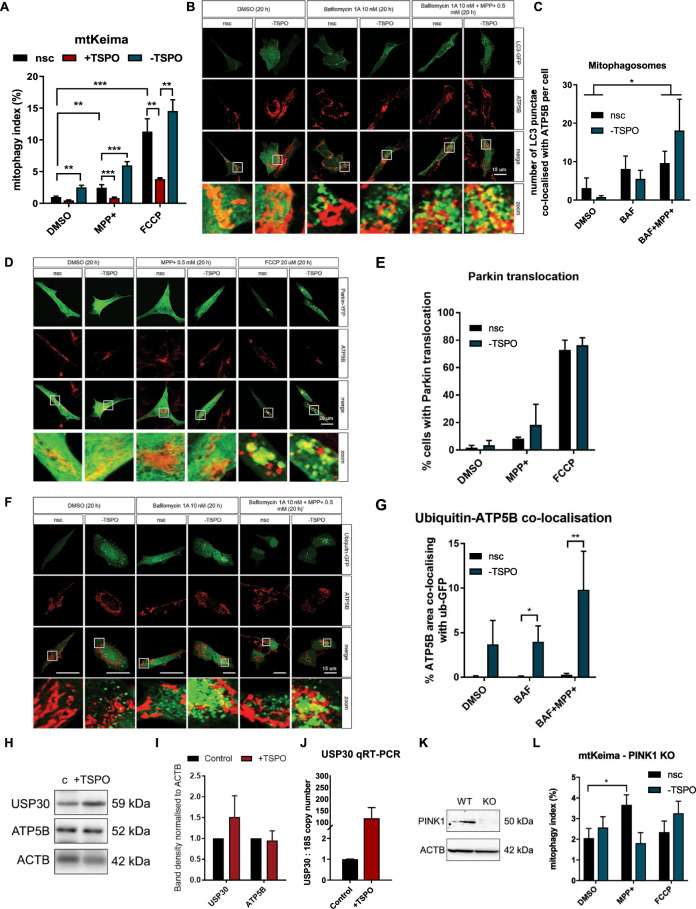
TSPO represses the MPP+ mediated activation of mitophagy. **A** Mitophagy index measured with mtKeima in mock-transfected, ±TSPO cells treated with vehicle (DMSO), 0.5 mM MPP+ or 20 µM FCCP for 20 h (*n* ≥ 20 cells). **B** ICC for ATP5B to label mitochondria (red) in cells transfected with TSPO siRNA/nsc and GFP-tagged LC3 and treated with either vehicle (DMSO), 10 nM Bafilomycin 1A and 0.5 mM MPP+ for 20 h. The average number of mitochondrial LC3 punctae quantified in (**C**) (*n* ≥ 10 cells). **D** ICC analysis of Parkin translocation to mitochondria, immunostained for ATP5B. -TSPO cells transfected with Parkin-YFP, were treated for 20 h with DMSO, 0.5 mM MPP+ or 20 µM FCCP. Percentage of cells with Parkin punctuation onto mitochondria (*n* ≥ 20 cells per condition) were represented in (**E**). **F** ICC for ATP5B to label mitochondria (red) in Control and -TSPO cells GFP-tagged ubiquitin and also treated with either vehicle (DMSO), 10 nM Bafilomycin 1A and MPP+ (0.5 mM, 20 h). **G** % of mitochondria co-localised with ubiquitin punctae was used a measure of mitochondrial ubiquitination. *n* ≥ 10 cells per condition. **H** Western blot of mock-transfected cells or overexpressing TSPO. Whole-cell protein lysates were probed for USP30 and a mitochondrial marker ATP5B. The blot was quantified in (**I**) (*n* = 3). **J** mRNA from Control and +TSPO cells was used for a quantitative reverse transcriptase PCR. The absolute mRNA copy number of USP30 was extrapolated and normalised to 18S mRNA copy number. Each +TSPO value was then normalised to its Control (*n* = 3). **K** Representative WB bands of whole-cell protein lysates of WT and PINK1 KO SH-SY5Y cells probed for PINK1 and β- actin (ACTB). **L** mtKeima measurement of mitophagy in nsc or −TSPO PINK1 KO cells treated with vehicle (DMSO), MPP+ (0.5 mM, 20 h) or FCCP (20 µM, 20 h). *n* ≥ 10 cells.

Mitophagy can function as a quality-control stress response to remove damaged mitochondria. It can be induced by a variety of toxic or non-toxic triggers [82] and be carried out by different pathways [83]. Within the context of neurodegeneration, the PINK1-Parkin pathway has been linked more strongly to pathology and studied at highest depth [7]. However, previous studies showed an activation of Parkin-independent mitophagy following neurotoxin addition [70, 71, 84, 85]. Therefore, we monitored the steps of the mitophagic cascade in response to MPP+. We began by enrolling the mtKeima reporter, which measures mitochondrial delivery to lysosomes, comparing Control, +TSPO, and −TSPO SH-SY5Y cells (Fig. 4A). TSPO downregulation induced a significant increase in mitophagy index both at resting conditions and after MPP+ treatment, while TSPO overexpression induced the opposite but less pronounced phenomenon. Oppositely, the mitochondrial uncoupler carbonyl cyanide-4-(trifluoromethoxy) phenylhydrazone (FCCP), commonly used to activate PINK1-Parkin-mediated mitophagy, induced a robust and comparable increase in mitophagy index in Control and -TSPO cells, but a strong reduction caused by TSPO overexpression. Notably, the MPP+induced increase in signal was quantified as being 30–50% that of FCCP-treated cells (Fig. 4A).

To confirm the involvement of the autophagy pathway in response to MPP+ treatment, we measured autophagosome and Parkin recruitment on mitochondria in SH-SY5Y cells expressing the fluorescent reporters LC3-GFP (Fig. 4B, C) and Parkin-YFP (Fig. 4D, E), calculated as a number of mitochondrial LC3 punctae, or mitophagosomes, per cell. Bafilomycin 1A (BAF) (10 nM), an inhibitor of the vacuolar-type H(+)-ATPase, was used to block lysosomal acidification and prevent autophagic degradation [86], therefore allowing the accumulation of the mitophagosomes. Both analyses demonstrated that MPP+ treatment does not block the translocation of Parkin on mitochondria. Therefore, in order to gain insights into the mechanism via which TSPO inhibits mitophagy during MPP+ treatment, we assessed the levels of mitochondrial ubiquitination in Control and TSPO SH-SY5Y cells by measuring the degree of co-localisation between ATP5B and ubiquitin. Notably, repression of TSPO activity increased basal levels of mitochondrial ubiquitination (Supplementary Fig. 3I–L), which were increased further when BAF was combined with MPP+ (Fig. 4F, G). Oppositely, TSPO over-expression, simulating the upregulation seen after treatment, increased the level of the deubiquitinase (DUB) ubiquitin specific peptidase 30 (USP30), as shown both via WB (Fig. 4H, I) and a qRT-PCR analysis (Fig. 4J). USP30 is the sole mitochondrially localised DUB [87] that impairs basal [88] and Parkin-mediated mitophagy [46]. Its inhibition has been proven to induce basal mitophagy upstream of PINK1, without inducing Parkin translocation [88]. To confirm the engagement of USP30 in the mitophagy pathway inhibited by TSPO, we transfected PINK1 KO SH-SY5Y cells (Fig. 4K) with mtKeima and measured the number of mitochondria removed by lysosomes in the presence and absence of TSPO (Fig. 4L). As expected, we observed an abolishment of the pro-mitophagic effects of TSPO interference, alongside the absence of any effect induced by FCCP.

Nevertheless, MPP+ generated a significant increase in the mitophagy index of control cells, indicating that, in the presence of TSPO, the neurotoxin induces mitochondrial delivery to lysosomes without engaging the PINK1-Parkin mitophagy pathway. Collectively, these data confirm a role for TSPO in basal and stress-induced mitochondrial quality control via de-ubiquitinating mechanisms. However, the necessity of utilising BAF to detect LC3 recruitment to mitochondria and MPP+induced ubiquitination implied a downstream effect on the autophagolysosomal pathway, which we therefore explored.

### Regulation of autophagolysosomal response by TSPO

**Fig. 5:**
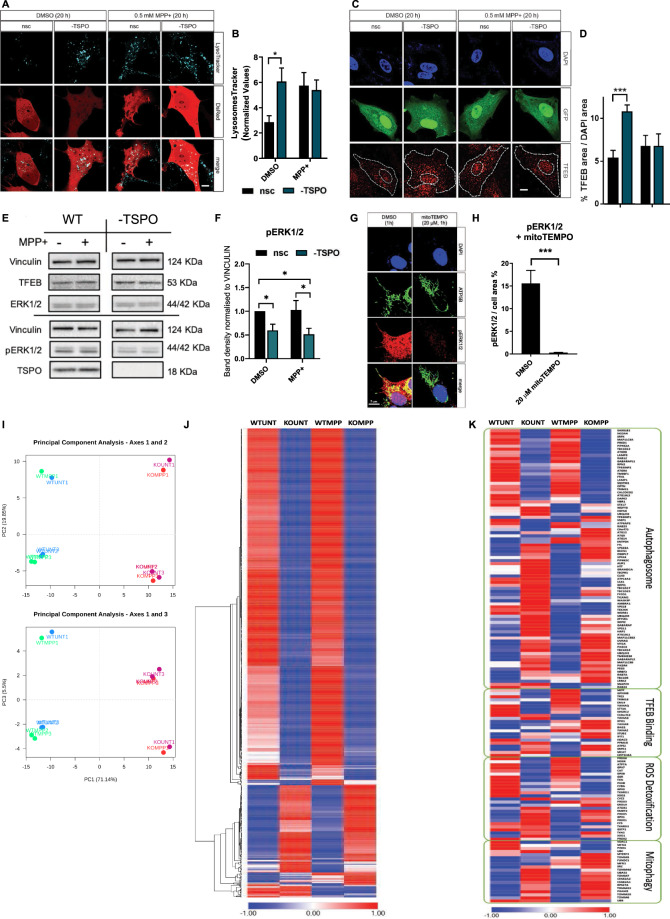
Lysosomal homeostasis is impaired by TSPO. **A** Representative images of LysoTracker staining of the lysosomal population in cells control or -TSPO and treated with vehicle (DMSO), 0.5 mM MPP + for 20 h. **B** Quantification of the total lysosomal number per cell (*n* ≥ 20 cells). **C** Cells transfected with GFP control (nsc) and −TSPO were immunostained for TFEB following treatment with vehicle (DMSO), or 0.5 mM MPP+ for 20 h. **D** Quantification of TFEB nuclear localisation (*n* ≥ 15 cells). **E** Representative bands of Vinculin, TFEB, TSPO, ERK1/2, and pERK1/2 from WB SDS-PAGE of whole-cell lysates of Control (wt) and -TSPO cells. Average band density of (**F**) pERK1/2 normalised to ERK1/2 and to respective Control (*n* = 3). Representative images of pERK1/2 ICC in wild type cells treated with 20 µM mitoTEMPO (**G**). **H** Quantification of ERK1/2 phosphorylation via image-based analysis of pERK1/2 area over cell area (*n* = 20 cells). **I** Principal component analysis of RNAseq data from Control and -TSPO cells. Heat maps in (**J** and **K**) are respectively of the full genome and genes involved in the cell’s quality control regulation.

**Fig. 6:**
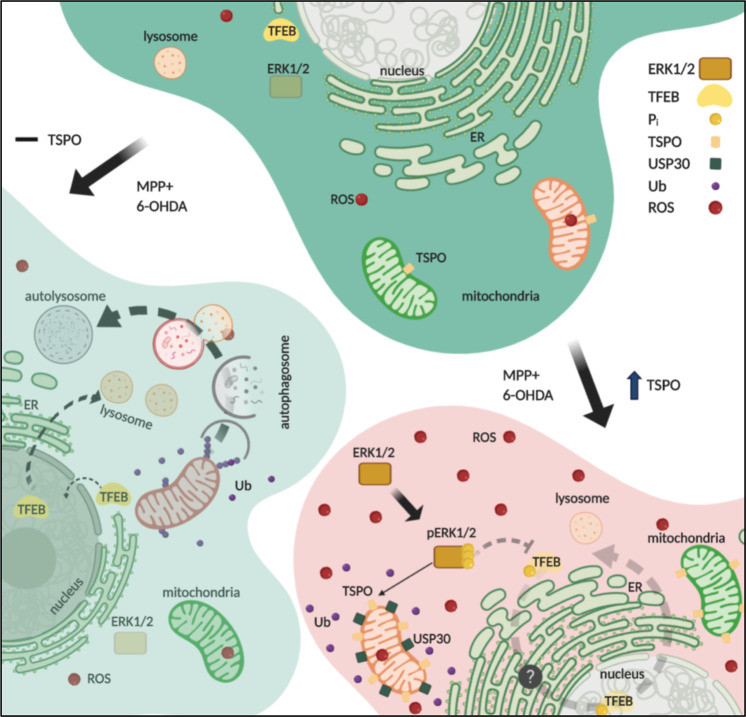
Model on TSPO role in the etiopathogenesis of neurotoxicity. The addition of PD neurotoxins like MPP+ or 6-OHDA stimulates TSPO upregulation, which, by accumulating USP30 on mitochondria, impairs the organelle’s ubiquitination mitophagy impairment. Concomitantly, it induces redox stress to promote ERK1/2 phosphorylation and modifications in the dynamics of the autophagolysosomal pathways.

Using the live-cell imaging dye LysoTracker™ DND-22 to mark lysosomes fluorescently, we assayed the lysosomal population in Control and −TSPO SH-SY5Y cells treated with MPP+ (0.5 mM, 20 h) (Fig. 5A, B). The results indicated that MPP+ induces an increase in lysosomal counts in control cells, which reached values comparable to those registered in −TSPO cells at resting conditions. However, this effect was lost when the MEK-induced phosphorylation of ERK1/2 pathway was inhibited via UO126. Since the microphtalmia/trascription factor E (MiT/TFE) family of transcription factors comprises the most important regulators of lysosomal turnover [89], we sought to test their involvement, and by using ICC, we assayed the levels and subcellular localisation of transcription factor EB (TFEB) [90–92] (Fig. 5C, D). In our experimental system, we found that in −TSPO cells, the amount of nuclear TFEB increases when compared to control ones, and so does the number of lysosomes and their volume (Supplementary Fig. 2G, H). Being the expression of TSPO driven by the activation of the ERK pathway, which is engaged by MPP+ [52] we explored whether the levels of TSPO affected the degree of pERK1/2 phosphorylation; the data in Fig. 5 panels E, F do confirm that TSPO does increase the degree of ERK1/2 phosphorylation. Having observed that MPP+ and TSPO increase global ROS levels (Fig. 3), we sought to determine whether this effect could modulate ERK1/2 phosphorylation. Protein kinase Cε (PKCε) is sensitive to redox status [93] and is known to phosphorylate ERK1/2 as well as to mediate phorbol myristate acetate (PMA)-induced *TSPO* upregulation [49, 94]. Therefore, TSPO may control its upstream regulator via a positive feedback mechanism, relying on oxidative stress. Furthermore, in keeping with this, the levels of pERK1/2 are dramatically reduced when TSPO-positive cells are treated with the antioxidant mitoTEMPO (Fig. 5G, H). In order to confirm this and corroborate the ample role played by TSPO on autophagy we mapped the transcriptome of dopaminergic neurons control (WT) versus those genetically depleted for TSPO (KO), untreated (UNT), or treated (MPP+) with the PD neurotoxin of interest. The next generation sequencing (NGS) analysis portraited a significant variance in the expression of the relative genes (Fig. 5I, J and Supplementary Fig. 4G, H) which was further evident among those which partake in general and selective autophagy (Fig. 5K). The working model depicted in Fig. 6 therefore proposes TSPO as required in the pathway to repress selection of mitochondria and therefore promote the aetiology of the whole network during neurotoxicity.

## Discussion

The role of mitochondria in PD is known for nearly half of a century [4, 32, 95], but our understanding of their contribution to the disease’s pathogenesis is unresolved. The mitochondrial decay in faulty dopaminergic neurons expands beyond the impairments in energy supply and second messengers (i.e. Ca^2+^) spanning inter-organelles communication and quality control mechanisms [7, 31, 96, 97]. This re-set the focus on mitochondrial aetiology of PD: 5 out of 18 risk factor genes for PD are linked to mitophagy (*PARK2, PINK1, DJ-1, FBXO7, HTRA2)*. The incapacity of removing defective mitochondria through the process of mitophagy reroutes functional and architectural homeostasis of neurons to deficiencies. Finding early markers of mitochondrial commitment to PD is therefore pivotal to pave the way to improved diagnostic and therapeutic protocols.

In this work, we demonstrate TSPO to be one of those. Though enrolled to map neuroinflammation in several diseases [22, 98], little is known of TSPO molecular function in the pathogenesis of the conditions in which it is found upregulated [99, 100], and in which its ligands mediate neuroprotection [101]. In mice injected with MPTP (the pro-drug catalysed by MAO into MPP+ [102]), treatment with XBD-173 (TSPO ligand) [103] preserves motor functions and striatal dopaminergic circuits [101]. Our data show that PD exploits TSPO to establish archetypical mitochondrial defects of the disease: increased redox stress (i), impaired mitophagy (ii), and susceptibility to dopamine-induced cell death (iii).

The heightened basal redox state in TSPO overexpressing neurons is not only due to the upregulation of the cytoplasmic NADPH oxidase [60], but also by the parallel accumulation of MAOB (Fig. 3H), which amplifies the presence of free radicals [104, 105].

Interestingly, the absence of an MPP+induced spike of ROS in +TSPO cells indicates that neurons rich in TSPO retain higher levels of reduced GSH, which is consumed following MPP+ administration (Supplementary Fig. 1E–G). Therefore, we speculate that the excess of GSH reacts with the additional ROS preventing further and detrimental burst. In +TSPO cells, the greater GSH level may originate from the inhibition of Keap1, which would allow for the Nuclear factor erythroid 2-related factor 2 (Nrf2) to migrate to the nucleus and upregulate the synthesis of GSH [106, 107]. The Nrf2 pathway is indeed responsible for the increased production of GSH s-transferase that executes the rate-limiting step in GSH production in the cytoplasm [108–110]. This was previously confirmed pharmacologically via the pro-mitophagic drug PMI which represses TSPO [111].

The ability of TSPO to increase redox signalling while leaving unaltered the antioxidant pools would also explain why +TSPO cells resist cell death caused by a higher concentration of MPP+ and excessive dopamine level (Fig. 2K, L) besides preventing the exacerbation of the MPP+induced oxidative stress (Fig. 3D, E, S). Accordingly, cells devoid of TSPO (−TSPO) undergo extensive ΔΨ_m_ depolarisation following treatment with MPP+ (Supplementary Fig. 2A–F), being at the same time more susceptible to death induced by the toxin (Fig. 2K) but not to the one caused by dopamine (Fig. 2L); indicative of an ongoing selection of defective mitochondria selection by mitophagy in these cells. And the analysis of mitochondrial morphology did reveal a more elongated and branched network of mitochondria in -TSPO cells corresponding to more favourable bioenergy [112, 113] as witnessed by the higher basal ΔΨ_m_ (Supplementary Fig. 2A–E).

Both 6-OHDA or MPP+ trigger the MAPK/ERK pathway, which drives the expression of TSPO. This establishes a feedback loop for which ERK is continuously active -by the high degree of oxidative stress- repressing the retro-translocation of TFEB to the nucleus and so the overall autophagy response by the repressed production of lysosomes (Figs. 4, 5).

Though deficiencies in lysosomal homeostasis could also be contributed by the impaired Ca^2+^ homeostasis driven by TSPO, the persistent level of free radicals of the oxygens in the cytosol (i) and the very nature of the trigger (MPP+) which engages the MAPK signalling (ii) suggest alterations in the TFEB transcriptional capacity to be the driving mechanism.

Nuclear TFEB, an essential transcription factor for the completion of the autophagolysosomal pathway [90, 114], is upregulated in -TSPO cells and during challenge with PD toxins (Fig. 5). This made us propose that MPP+ {and therefore the neurotoxins capable of recapitulating the disease [68, 69, 115]} activate mitophagy by stimulating TFEB, which is negatively regulated by the concomitant overexpression of TSPO (Fig. 5). The transcriptome data well corroborate that loss of TSPO in neurons is pivotal to several genetic alterations, which are exploited further by the treatment with the neurotoxin MPP+ (Fig. 5 and Supplementary Fig. 4G, H). The limited availability of autophagosomes and production of lysosomes (Fig. 5) aligns with the deubiquitylation of mitochondria perpetrated by TSPO.

Mitophagy occurs if de-ubiquitinating molecules such as USP30 are inhibited [88]. TSPO, on the contrary, stabilises USP30, thereby impeding the processing of mitochondrial proteins required for the completion of the pathway (Fig. 4H, I). USP30 has been considered a therapeutic target since the discovery of its involvement in mitophagy [46], whereas its inhibition rescues motor symptoms and lifespan of PD *Drosophila* models. The recent publications of three proteomic studies [44, 45, 116] have highlighted that changes in the ubiquitylome at appropriate dosages faithfully replicate the genetic ablation of USP30, which nonetheless remains particularly hostile to target with chemicals. TSPO could therefore offer an alternative site for therapeutic inferring of USP30, and advantages for this approach stem from the extensively studied pharmacodynamic and pharmacokinetic properties of its ligands [17, 20, 117].

It is known that the monogenic forms of the disease constitute only 5–10% of the total PD cases [118, 119], demanding for a greater understanding of the aetiological factors at the basis of the sporadic forms, which account for the greatest portion of cases. TSPO merges as a potential target to inform and control both onset and penetrance. Though its anti-mitophagic role will intensify the retro-communication with nucleus [120] to favour cell survival, this beneficial effect will be short-lived as TSPO-positive neurons will succumb to dopamine toxicity in the long-term (Fig. 2L and Supplementary Fig. 4A–F).

Importantly the upregulation of TSPO in response to neurotoxins is selective for neurons as microglial cells are unresponsive to PD toxins (Supplementary Fig. 3A, B).

In light of these data, it is not surprising that direct detection of the protein in mice and in vivo PET measurements demonstrate higher TSPO expression with aging [60, 121], which is per se a demonstrated risk factor for the development and progression of Parkinson’s disease (PD).

## Supplementary information

Supplementary Material
