# Supplementary material for: The translocator protein (TSPO) is prodromal to mitophagy loss in neurotoxicity
Source: Mol Psychiatry. 2021 Mar 4;26(7):2721–39. doi: 10.1038/s41380-021-01050-z (PMC8505241; doi:10.1038/s41380-021-01050-z)
Supplement: Supplementary file 1 — Supplementary Material [file 41380_2021_1050_MOESM1_ESM.pdf]

Supplementary Figure 1

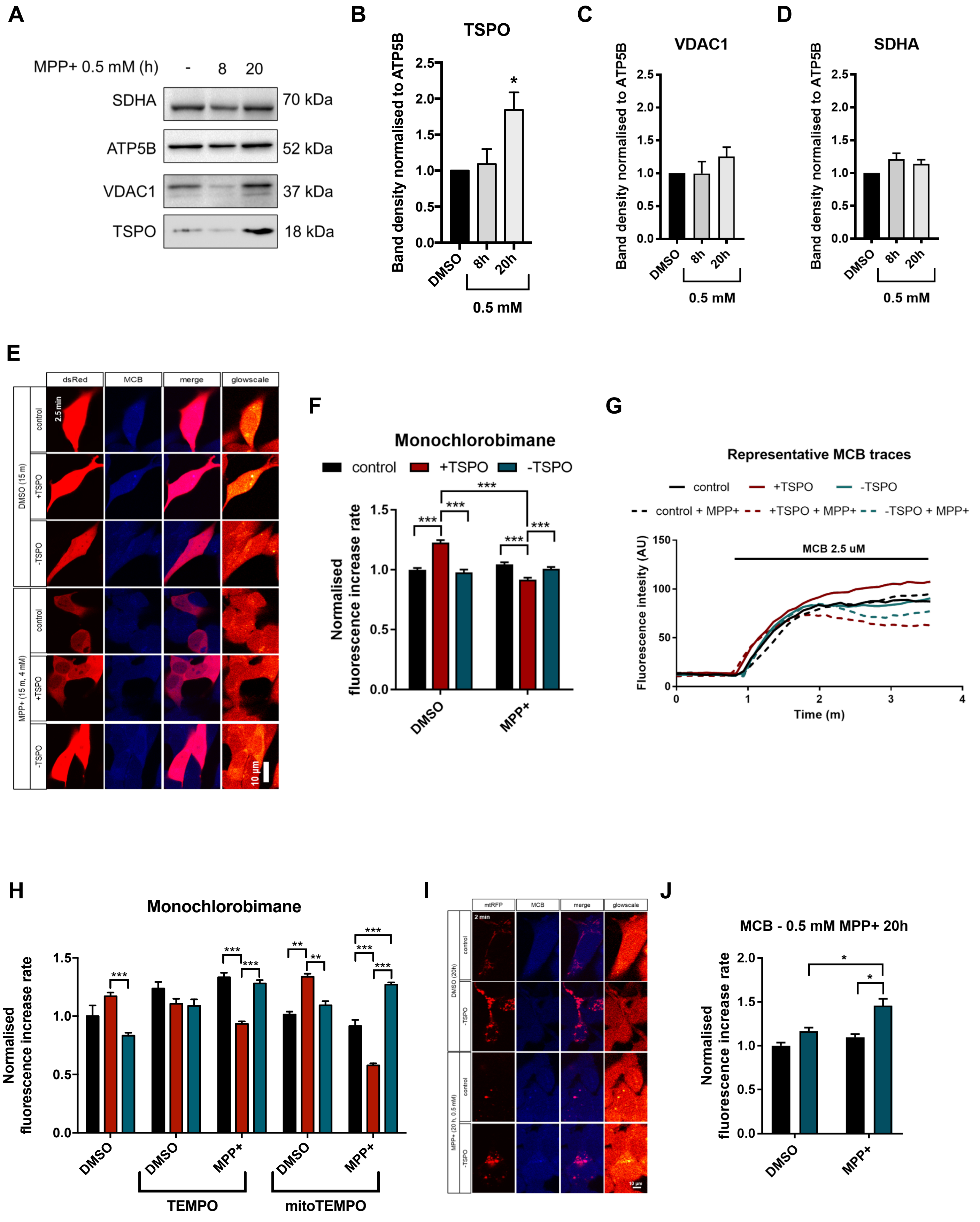

Supplementary Figure 2

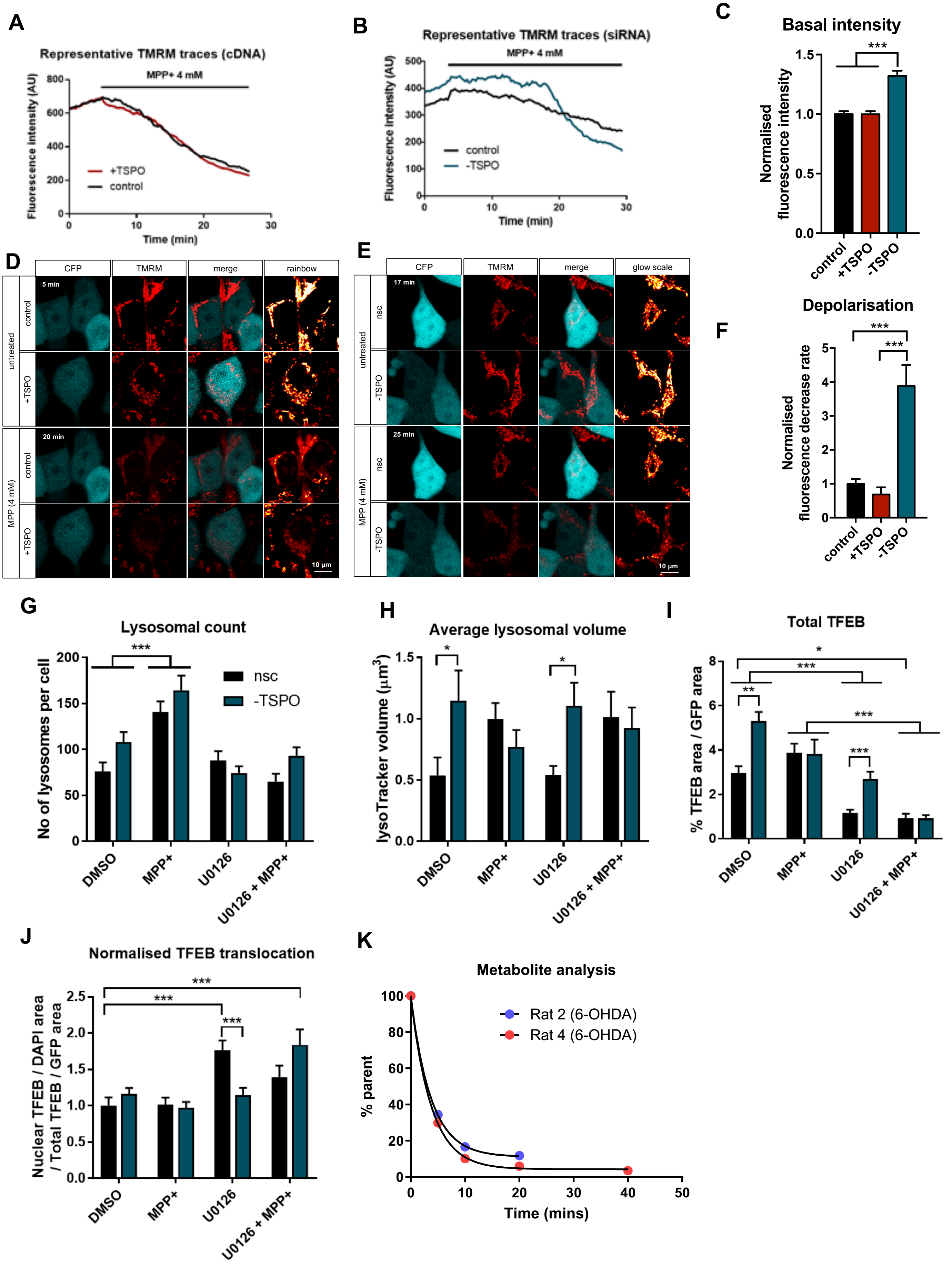

Supplementary Figure 3

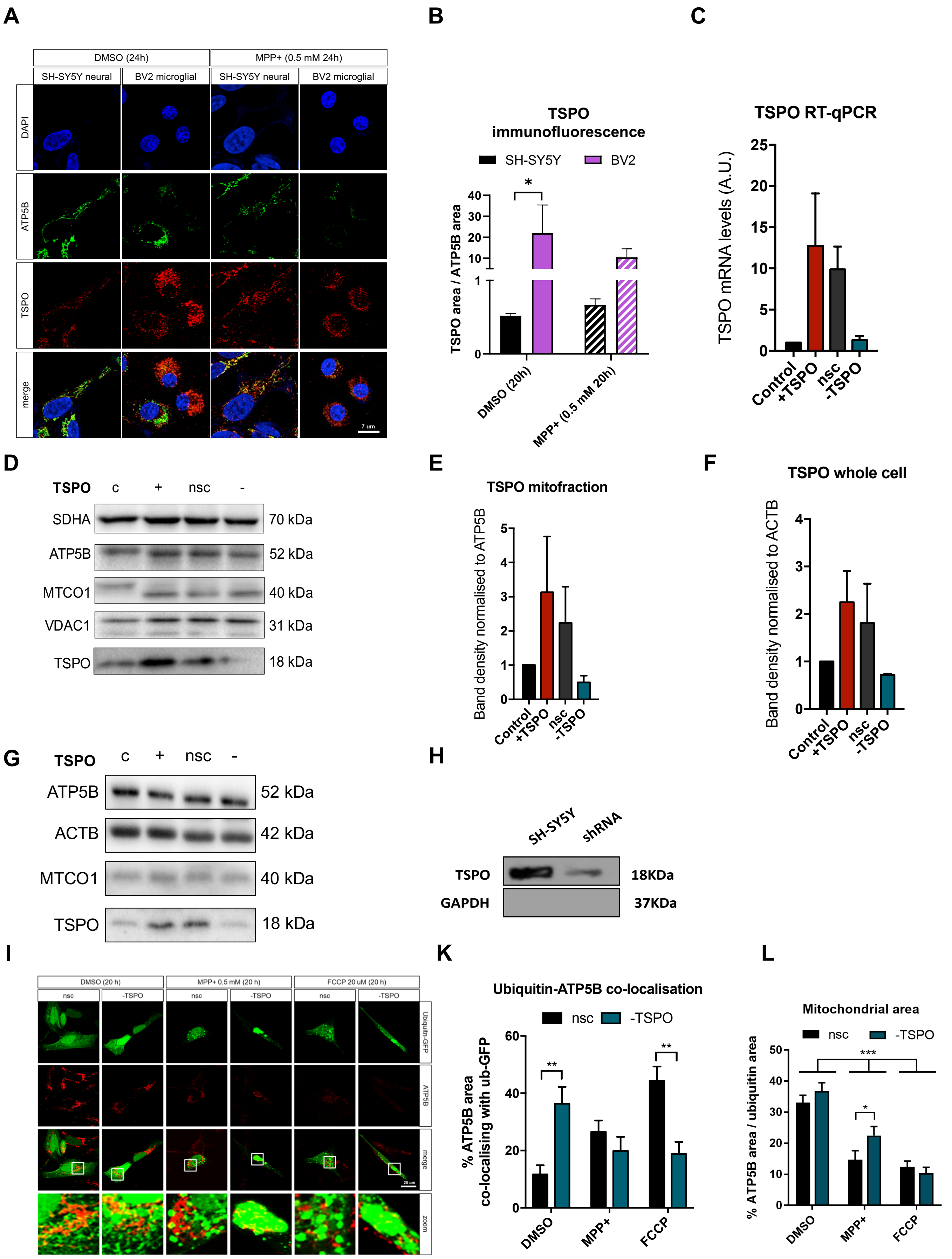

Supplementary Figure 4

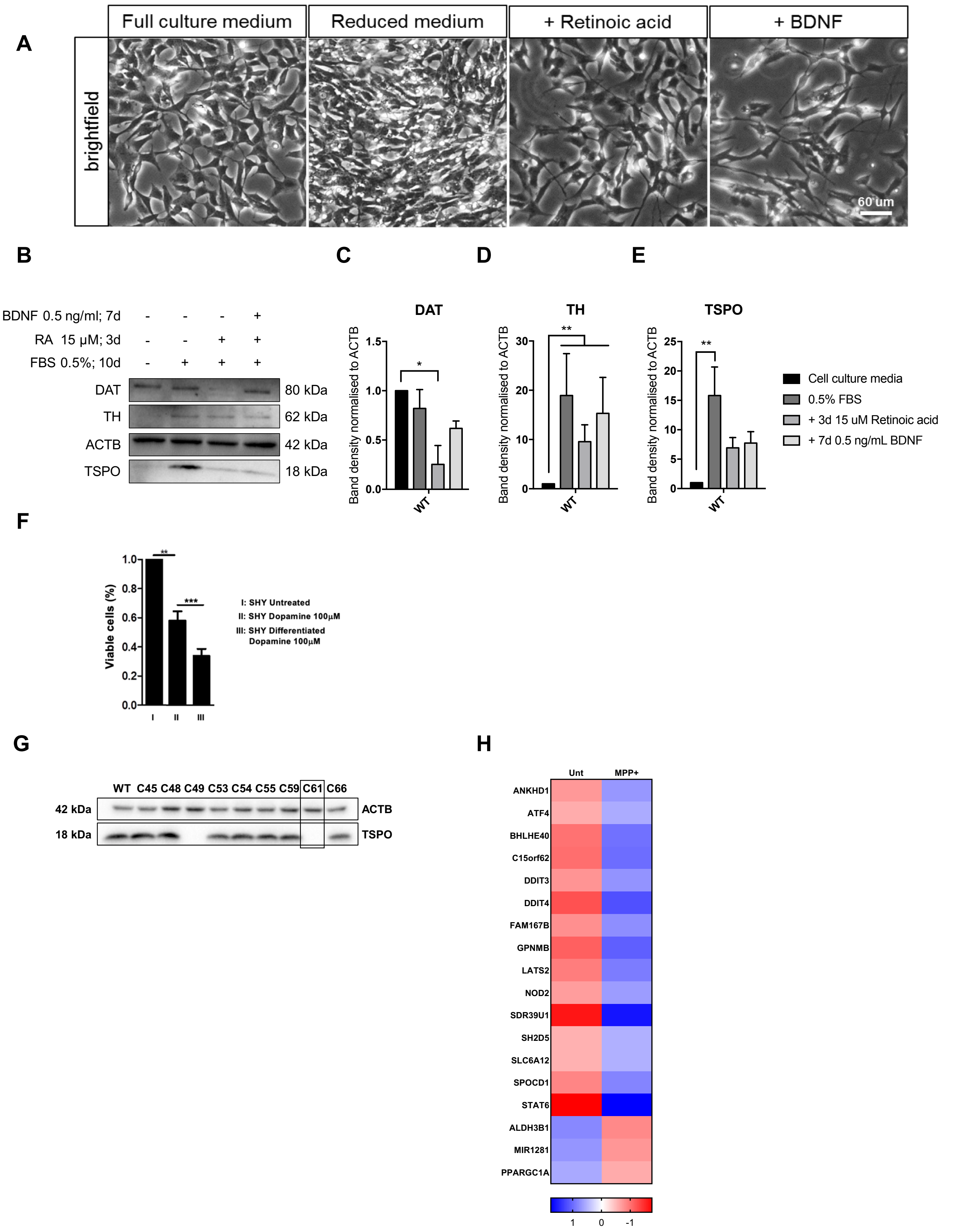

Legends to Supplementary Figures

Supplementary Figure 1. (**A**) Immunostained bands from mitochondrial fraction analysis via WB of cells treated with 0.5 mM MPP+ for 0, 8 or 20 hours. Band density quantification for (**B**) TSPO, (**C**) VDAC1 and (**D**) SDHA normalised to ATP5B (n≥3). (**E**) Representative images from MCB live staining of cellular free reduced glutathione in control and +/- TSPO cells co-transfected with the DsRed reporter and pre-treated with DMSO or 4 mM MPP^+ for 15 minutes, with (**F**) quantification of average whole cell fluorescence normalised to control (n≥20 cells) and (**G**) representative curves (**H**) MCB Quantification of reduced cellular GSH following acute addition of vehicle or 4 mM MPP+ (15 min), with addition of pharmacological quenching of mitochondrial and cytoplasmic ROS separately, using mitoTEMPO and TEMPO (n≥10 cells). (**I**) Representative images of MCB staining of cells pre-treated with sub-lethal MPP^+ following transfection with mtRFP and TSPO RNAi, with quantification in (**J**) (n≥15 cells). All data is shown as mean ± SEM. '*' – p<0.05, '**' – p<0.01, '***' – p<0.001.

Supplementary Figure 2. (**A**) Representative TMRM traces of mock transfected and TSPO-overexpressing cells, during live recording of MPP+ addition (4 mM MPP+). (**B**) Representative TMRM images of cells transfected with CFP, co-transfected with TSPO scrRNA/siRNA and treated acutely with 4 mM MPP+. (**C**) Basal mitochondrial membrane potential, $\Delta\psi_m$, of +/- TSPO cells (**D**) Representative TMRM images of cells transfected with CFP or co-transfected with TSPO-expressing plasmid and treated acutely with 4 mM MPP+. (**E**) Representative TMRM images of nsc/-TSPO cells with 4 mM MPP^+ during live recording and (**F**) rate of $MPP(^+)$-induced $\Delta\psi_m$ depolarisation (4 mM, acute) (n=20 cells). (**G**) Quantification of lysosomal count per cell and (**H**) average volume of lysosomes via LysoTracker blue in cells transfected with TSPO RNAi and treated with vehicle, 10 μM U0126 and/or 0.5 mM MPP+ for 20 hours (n≥20 cells). (**I**) Cells transfected with GFP and TSPO RNAi or control were immunostained for TFEB following treatment with vehicle, 10 μM U0126 and/or 0.5 mM MPP+ for 20 hours. Quantification of total TFEB is given (n≥15 cells), as well as (**J**) normalised TFEB translocation (n≥15 cells). (**K**) [^{11}C]PBR28 metabolite analysis in the plasma of two 6OHDA lesioned rats, as control for neuroimaging. All data is shown as mean ± SEM. '*' – p<0.05, '**' – p<0.01, '***' – p<0.001.

Supplementary Figure 3. (**A**) Neural SH-SY5Y cells and microglial BV2 cells were separately incubated with 0.5 mM MPP^+ for 20 hours and levels of TSPO and ATP5B, proxy of mitochondria, were measured by immunofluorescence. (**B**) Quantification of TSPO fiuorescence intensity as area above threshold normalised to area above threshold of ATP5B signal ($n \geq 40$ cells). (**C**) To alter levels of TSPO, co-transfection of a fluorescent protein (c) with either a TSPO plasmid (+), a scrRNA (nsc) or a TSPO siRNA (-) was used. The effectiveness of transfection was quantified by qRT-PCR. (**D**) Representative WB bands of mitochondrial fractions from samples transfected for TSPO showing various mitochondrial membrane proteins as well as TSPO, with its band density quantification in (**E**), normalised to ATP5B (n=3). (**F**) Average band density quantification of TSPO expression in whole cells lysates (n=3). (**G**) SDS-PAGE of whole cell lysates from SH-SY5Y cells transfected for TSPO with loading control (ACTB) and two mitochondrial proteins, ATP5B and MTCO1. (**H**) Representative WB of SH-SY5Y cells with a constitutive insertion of TSPO-targeting shRNA, decreasing its expression with regards to loading control GAPDH. **.** (**I**) Representative images of ICC of nsc/-TSPO cells transfected with ubiquitin-GFP and treated for 20 hours with DMSO, 0.5 MPP+ or 20 μM FCCP and analysed for (**K**) ubiquitin-ATP5B co-localisation and (**L**) mitochondrial area ($n \geq 30$ cells).

Supplementary Figure 4. SH-SY5Y cells were treated with reduced medium for 10 days, with addition of RA for the first three days and successively substituting it for BDNF. The contribution of the different steps to differentiation, and their characterisation, is shown above by: (**A**) morphological comparison of brightfield images of live cells; (**B**) western blotting to show expression of neuronal markers (**C**) DAT, (**D**) TH and TSPO in (**E**) (n=4). (**F**) Comparison of viability, measured by Trypan blue, of undifferentiated and differentiated SH-SY5Y cells treated with 100 μM Dopamine. (**G**) Western blotting analysis of SH-SY5Y cells edited for TSPO ablation via CrisprCas9 vector. (**H**) Heatmap analysis for the overexpression of TFEB regulated genes in cells exposed to MPP^+.
